# BDdb: a comprehensive platform for exploration and utilization of birth defect multi-omics data

**DOI:** 10.1186/s12920-021-01110-x

**Published:** 2021-11-04

**Authors:** Dengwei Zhang, Si Zhou, Ziheng Zhou, Xiaosen Jiang, Dongsheng Chen, Hai-Xi Sun, Jie Huang, Shoufang Qu, Songchen Yang, Ying Gu, Xiuqing Zhang, Xin Jin, Ya Gao, Yue Shen, Fang Chen

**Affiliations:** 1 College of Life Sciences, University of Chinese Academy of Sciences, Beijing, 100049 People’s Republic of China; 2 BGI-Shenzhen, Shenzhen, 518083 People’s Republic of China; 3 Guangdong Provincial Key Laboratory of Genome Read and Write, BGI-Shenzhen, Shenzhen, 518120 People’s Republic of China; 4 Shenzhen Institute of Synthetic Biology, Shenzhen Institutes of Advanced Technology, Chinese Academy of Sciences, Shenzhen, People’s Republic of China; 5 BGI Genomics, BGI-Shenzhen, Shenzhen, 518083 People’s Republic of China; 6 China National Genebank, BGI-Shenzhen, Shenzhen, 518120 People’s Republic of China; 7 National Institutes for Food and Drug Control (NIFDC), Beijing, 100050 People’s Republic of China; 8 Guangdong Provincial Key Laboratory of Human Disease GenomicsShenzhen Key Laboratory of Genomics, BGI-Shenzhen, Shenzhen, 518083 People’s Republic of China; 9 Shenzhen Engineering Laboratory for Birth Defects Screening, BGI-Shenzhen, Shenzhen, 518083 People’s Republic of China; 10 MGI, BGI-Shenzhen, Shenzhen, 518083 People’s Republic of China

**Keywords:** Birth defects, Chromosomal abnormality, Omics, Biomarker

## Abstract

**Background:**

Birth defects pose a major challenge to infant health. Thus far, however, the causes of most birth defects remain cryptic. Over the past few decades, considerable effort has been expended on disclosing the underlying mechanisms related to birth defects, yielding myriad treatises and data. To meet the increasing requirements for data resources, we developed a freely accessible birth defect multi-omics database (BDdb, http://t21omics.cngb.org) consisting of multi-omics data and potential disease biomarkers.

**Results:**

In total, omics datasets from 136 Gene Expression Omnibus (GEO) Series records, including 5245 samples, as well as 869 biomarkers of 22 birth defects in six different species, were integrated into the BDdb. The database provides a user-friendly interface for searching, browsing, and downloading data of interest. The BDdb also enables users to explore the correlations among different sequencing methods, such as chromatin immunoprecipitation sequencing (ChIP-Seq) and RNA sequencing (RNA-Seq) from different studies, to obtain the information on gene expression patterns from diverse aspects.

**Conclusion:**

To the best of our knowledge, the BDdb is the first comprehensive database associated with birth defects, which should benefit the diagnosis and prevention of birth defects.

**Supplementary Information:**

The online version contains supplementary material available at 10.1186/s12920-021-01110-x.

## Background

Birth defects refer to abnormalities present at birth in form, function, biochemistry, and mentality [1]. More than 8.14 million children are born with severe birth defects each year, which are among the principal causes of infant mortality [2]. The impacts of birth defects on human society are widespread, not only affecting survival and quality of life for those affected, but also resulting in emotional and economic burdens on the family [3]. Although many studies have attempted to unveil the causes of birth defects, most remain vague [2, 3]. The established causes of birth defects can be roughly divided into three categories: i.e., genetic factors, environmental factors, and their interactions [4]. Genetic factors include chromosomal aberrations and genetic mutations, which can render severe intellectual disabilities and deformity [2, 5].

Chromosomal abnormalities are one of the main causes of birth defects with a known etiology [3], with nearly one in 200 newborns affected [6]. Most of these children suffer severe intellectual disability and tissue and organ deformities. The most common chromosomal abnormalities include trisomy 21 (Down syndrome), trisomy 18 (Edwards syndrome), trisomy 13 (Patau syndrome), and sex chromosome aneuploidies such as monosomy X (Turner syndrome), conferring more than 80% of prenatal diagnosis of chromosomal abnormalities [6]. Among them, Down syndrome is the most common disease, with an occurrence of 1/319 to 1/1 000 in different populations [3, 7].

Birth defects resulting from chromosomal abnormalities have almost no effective treatment, although they are identifiable at early pregnancy through prenatal screening and diagnosis. To facilitate the study of human diseases, many databases (e.g., MalaCards, DisGeNET, DGA, RareDDB, miR2Disease, HEDD) have been developed with a focus on the annotation of diseases and related genes [8–13]. However, there is currently no multi-omics database for collating, storing, integrating, and displaying birth defect-related research datasets. To fill this gap, we developed a freely accessible birth defect multi-omics database (BDdb, http://t21omics.cngb.org).

The current version of the BDdb includes a total of 326 records derived from 136 GSE Series records, involving 12 tissues and 35 cell types for human, as well as 12 tissues and 17 cell lines for mouse. This database provides an interactive platform that allows quick retrieval of datasets of interest with pertinent analysis. Furthermore, the BDdb includes 869 manually curated biomarkers from six species, involving 22 birth-defects. In summary, the BDdb provides a comprehensive resource for researchers and clinicians.

## Construction and content

### Data collection

**Fig. 1:**
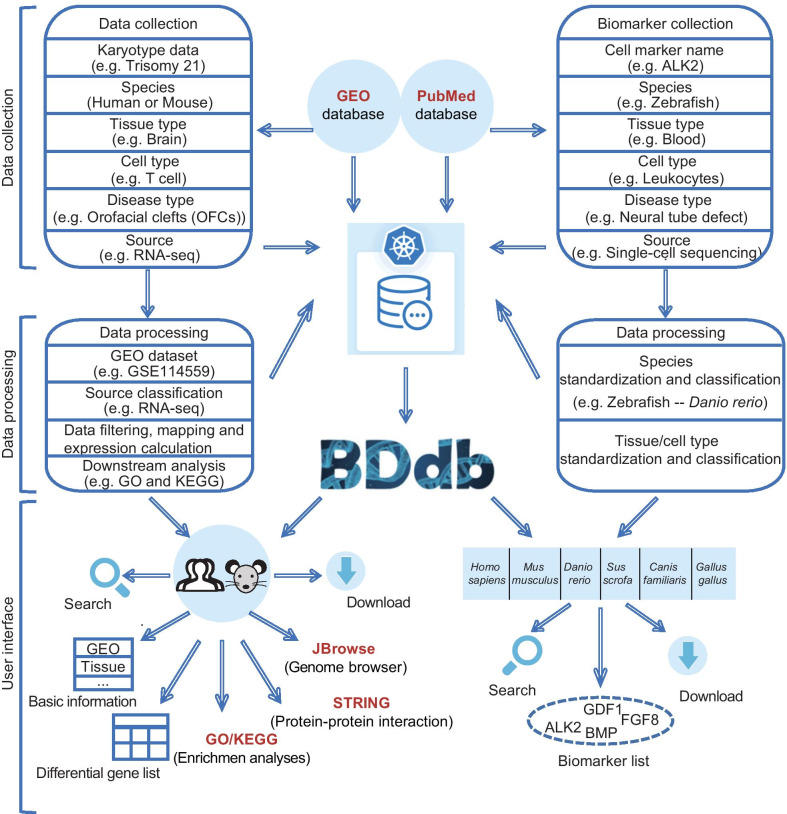
Overview of data collection, processing and database interface

The datasets used to build the BDdb were collected from the Gene Expression Omnibus (GEO, https://www.ncbi.nlm.nih.gov/geo/) database [14]. We first employed a list of keywords such as “trisomy 21”, “trisomy 13”, “trisomy 18”, “trisomy 8”, “monosomy X”, and “XXY” to retrieve pertinent information on *Homo sapiens* and *Mus musculus*, resulting in 328 GSE Series records (Fig. 1). All datasets were collected before August 2019. We then manually selected the relevant series records by careful reading of the retrieved data. Eventually, a total of 136 GSE Series records were selected and re-analyzed.

Single-cell RNA sequencing (scRNA-seq) holds tremendous potential for studying cell phenotype and cell behavior at single-cell resolution [15]. As such, we considered single-cell sequencing studies focusing on birth defects. We searched birth-disease related scRNA-seq datasets in humans and mice from the Sequence Read Archive (SRA) and GEO databases. We found one GSE Series (GSE127257) record [16], which included 13,766 cells. This dataset focused on the Ts65Dn mouse model for Down syndrome.

To obtain putative biomarkers associated with different birth defects from published studies, we searched PubMed using keywords, including disease names with “marker(s)”, “gene(s)”, and “genetic(s)”. More than 3 000 publications were obtained. We manually surveyed the abstracts of these publications, and if they were considered to include pertinent information, we downloaded the paper and its supplementary materials. We then manually checked the full text of the selected publications to obtain biomarkers with reliable evidence and gain useful information, including the title of the paper, PMID, disease type, sequencing type, tissue type, cell type, and species. Among the final results, more than 500 relevant publications were included, and marker gene lists were manually extracted.

### Data processing

For each dataset, we carefully read the original paper if available. When a dataset contained different karyotypes or models (or different types of sources, i.e., tissues and cell lines), we manually divided it into multiple sub-datasets in the BDdb (Fig. 1), with 326 (sub)-datasets thus generated (Additional file 1: Table S1).

Raw data from next-generation sequencing and microarrays were downloaded. These data were re-analyzed using a uniform pipeline due to the lack of complete analysis results. Some microarray datasets were difficult to re-analyze or contained problems. Thus, we used the integrated GEO2R tool provided by GEO website to obtain a list of differentially expressed genes (DEGs). Detailed analyses are as follows.

#### RNA-Seq

Raw reads were filtered by SOAPnuke [17] with the parameters “−l 15 −q 0.2 −n 0.05”. Clean reads were then aligned to human (GRCh38.p12, downloaded from GENCODE [18]) or mouse (GRCm38.p6, downloaded from GENCODE [18]) reference genomes using HISAT2 [19]. StringTie [20] was used to compute gene expression. To screen DEGs, DESeq2 [21] was adopted for samples containing replicates; otherwise, DEGs were obtained using edgeR [22]. DEGs were screened based on absolute log_2_-fold-change >  = 0.5, *p* < 0.05, and false discovery rate (FDR) < 0.1. Gene Ontology (GO) enrichment and Kyoto Encyclopedia of Genes and Genomes (KEGG) annotation were performed using clusterProfiler [23].

#### DNA methylation

Low-quality reads from Bisulfite sequencing (BS-Seq) or reduced-representation bisulfite sequencing (RRBS-Seq) were filtered with Trim Galore [24]. Subsequently, alignment of reads and extraction of methylation information were performed using Bismark [25]. Polymerase chain reaction (PCR) duplications were removed for the BS-Seq samples but not for RRBS-Seq sample as per the Bismark instructions. The R package methylKit [26] was used to extract the differential methylation regions among diverse samples.

#### DNA–protein interactions

An identical pipeline was adopted for analysis of chromatin immunoprecipitation sequencing (ChIP-Seq) and DNase I hypersensitive sites sequencing (DNase-Seq). SOAPnuke [17] was employed to remove low-quality reads, with the resulting clean reads then mapped against the human or mouse genome references using Bowtie2 [27]. Peak calling was performed using MACS2 [28, 29] and peak annotation was accomplished using CHIPseeker [30]. Lastly, DiffBind [31] was used to identify differential peaks.

#### Small RNA (smRNA) sequencing

Trim Galore [24] was adopted to trim the adaptors of the raw reads. Only smRNA reads that ranged in length from 18 to 30 nt were retained, with the parameters “–quality 20 –gzip –small_rna –max_length 30”. Eligible reads were then aligned against the human reference genome (GRCh38.p12, downloaded from GENCODE) and the miRBase [32] (release 22.1, http://www.mirbase.org/) to be annotated as miRNA, tRNA, rRNA, snoRNA, snRNA, piRNA, or other non-coding RNAs.

#### Microarray

For DNA microarray datasets with raw signal files, an in-house bioinformatics pipeline was adopted for quality control and expression quantification. Briefly, we read the *cel* file by using the R package affy (v1.8 release) [33], then, used the R packages simpleaffy [34] and affyPLM [35] to assess chips quality. Low-quality chips (actin3/actin5 > 3, gapdh3/gapdh5 > 1.25 and non-detected BioB) and *lowvar* probe sites were excluded. We next adopted the “rma” method for pretreatment. The expression abundance values of genes, including protein-coding genes (PCGs) and long non-coding RNAs (lncRNAs), were summarized using *t*-tests. Expression values were log_2_ transformed with an offset of 1. We resolved the up- and down-regulated genes, and then performed KEGG annotation and GO enrichment analysis with clusterProfiler [23]. Finally, we constructed a network of DEGs using STRING [36].

#### sci-ATAC-seq

We extracted corresponding metadata, including cell type and source (i.e., tissue, cell line). We also obtained the cell groups (referring to patient ID, culture condition or cell phenotype) from the supplementary tables of the papers and labeled each cell with the cell group information. Marker genes were then collected.

### Website construction

The website was constructed using CodeIgniter, a powerful PHP framework. CodeIgniter provides an Application Programming Interface (API) for connecting the website to the MySQL database. We also used JavaScript libraries, including jQuery (3.4.1), jQuery-Labelauty, and additional plugins, to perform dynamic web services.

### Data access

The BDdb website is accessible to users at http://t21omics.cngb.org, and offers a concise, well-organized interface. Users are welcome to add comments regarding their requirements and suggestions to improve the database. All provided data can be downloaded. The BDdb will be continually updated with additional data from pertinent research. Figure 1 shows the overall features of the database.

## Utility and discussion

### BDdb statistics

**Fig. 2:**
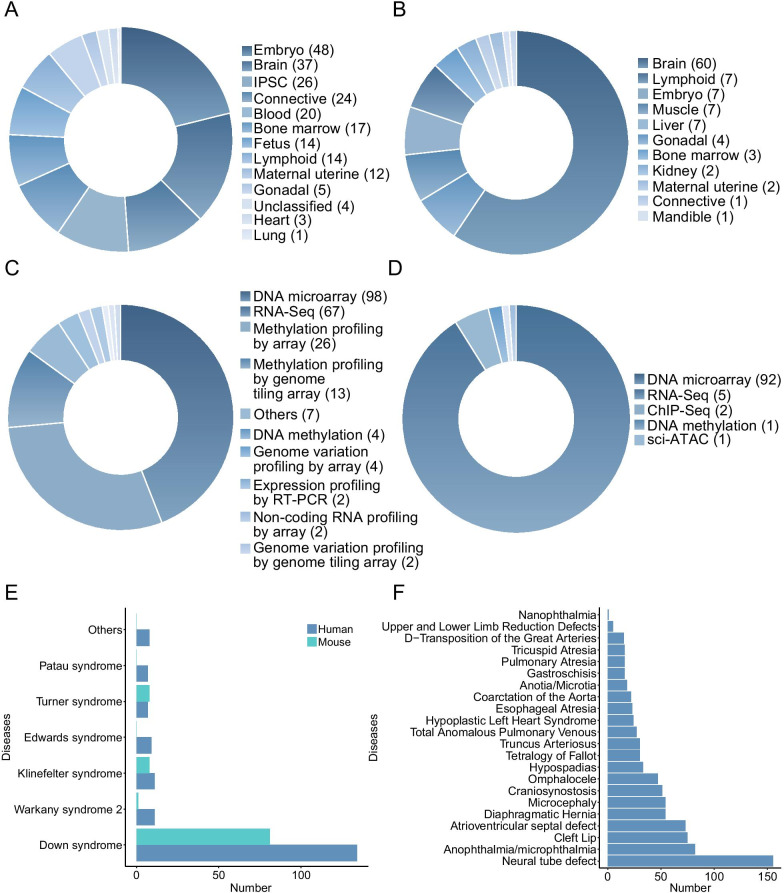
Statistics of datasets in BDdb. **a** Distribution of omics datasets for different tissue types in human. **b** Distribution of omics datasets for different tissue types in mouse. **c** Distribution of omics datasets for different sequencing methods in human. **d** Distribution of omics datasets for different sequencing methods in mouse. **e** Distribution of omics datasets for different diseases in human and mouse. **f** The numbers of datasets for different diseases regarding biomarkers. The numbers in brackets represent the number of datasets belonging to the corresponding tissues or sequencing methods

At present, the BDdb contains 101 and 37 GSE Series records from humans and mice, respectively. The obtained datasets include 15 diseases, 12 tissues, and 35 cell lines in humans, and four diseases, 12 tissues, and 17 cell lines in mice (Fig. 2a–e) obtained from multi-omics studies, e.g., genomics, transcriptomics, epigenomics, and single-cell omics (Fig. 2c, d). Moreover, the BDdb contains 869 potential biomarkers pertinent to 22 types of birth defects, such as microcephaly and neural tube defects (Fig. 2f). These markers were obtained from more than 500 studies involving six species, i.e., *Homo sapiens*, *Danio rerio*, *Mus musculus*, *Sus scrofa*, *Canis familiaris*, and *Gallus gallus*.

In the BDdb, embryonic tissues are among the most abundant samples for humans, followed by brain and blood; brain tissue samples are among the most abundant for mice, followed by lymphoid and embryonic samples (Fig. 2a, b). Correspondingly, the most abundant cell lines are blastocysts belonging to human embryo tissue and cortex belonging to mouse brain tissue. In terms of the diversity of sequencing types, DNA microarray data are dominant for both humans (44%) and mice (91%), followed by RNA-Seq, methylation profiling by array, ChIP-Seq, and DNA methylation (Fig. 2c, d). Most datasets are linked to Down syndrome, followed by Klinefelter syndrome, Turner syndrome, Warkany syndrome 2, and Edwards syndrome (Fig. 2e). In addition to datasets of diseases associated with chromosomal abnormalities, those related to diseases such as orofacial clefts, and open myelomeningocele are also included. For biomarkers collection, the top five diseases regarding related datasets include neural tube defects, anophthalmia/microphthalmia, cleft lip, atrioventricular septal defects, and diaphragmatic hernia (Fig. 2f). A summary of these datasets and biomarkers can be found in Additional files 1 and 2: Tables S1 and S2, respectively.

### Database features and utility

**Fig. 3:**
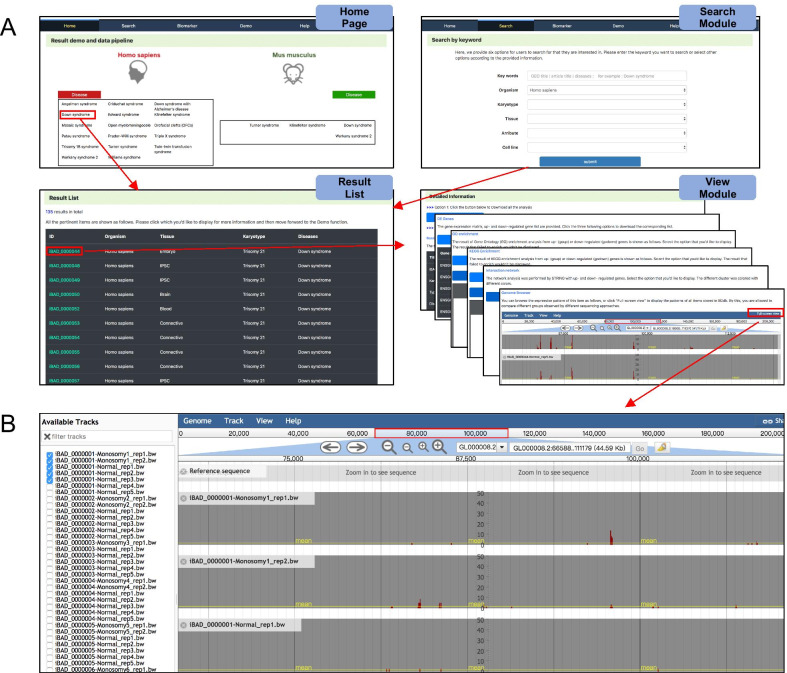
Screenshots of BDdb’s web interface. **a** An overall workflow in BDdb. Users can search the items through either home page or search module, and corresponding results would be displayed. **b** The Genome Browser interface, which enables users to interactively visualize genomic data from different studies

The BDdb contains multi-omics datasets and allows users to query the subsequent analysis results with five functional states. The easy-to-use interface provides access for searching, browsing, visualizing, and downloading (Fig. 3). The online user guide illustrates several cases of BDdb usage.

### Information search

For the search module, users can search by inputting keywords or choosing the provided options. As shown in Fig. 3a, users can select one or more options, including the organism, karyotype, tissue, and cell line. This enables users to opt for certain kinds of karyotypes, such as trisomy 21, trisomy 18, and monosomy X, which are typical for chromosome aneuploidy. In terms of omics datasets, the BDdb only contains those related to humans and mice at present. After submitting the search request, relevant results are displayed.

### View module

Data from diverse sequencing types are displayed with different result modules (Fig. 3a). For example, in the RNA-Seq datasets, the resulting interface contains five sections: (1) “Basic Information”, shows the information on karyotype, disease, organism, tissue, and cell line, which are the sample’s features, as well as the GEO title, literature, and searching link, which can help users to trace the origin of the data; (2) “DE Genes”, enables users to search or download the gene expression matrix as well as up- and down-regulated gene tables; (3) GO and KEGG enrichment, enables users to explore functional/pathway enrichment, with the bubble charts, bar charts, and cnetplots provided; (4) Network analysis of DEGs; (5) “Genome Browser”, can intuitively display expression patterns with a graphical interface. Apart from the RNA-Seq datasets, the database also provides fundamental analysis for other datasets. Particularly, omics datasets from different studies can be displayed in the “Genome Browser” as per the user’s requirements to further mine for useful information (Fig. 3b).

### Birth-defect diseases biomarker mapper

Biomarkers are stored in the “Biomarker” module. Users can search and view markers of interest by selecting species, diseases, and tissues from the pull-down menu. Users can also download all analysis and biomarker results via the ‘Download’ function. The BDdb also provides detailed tutorials and answers to common questions on the “Help” page.

### Case study: exploring biomarkers for diseases diagnosis using BDdb

**Fig. 4:**
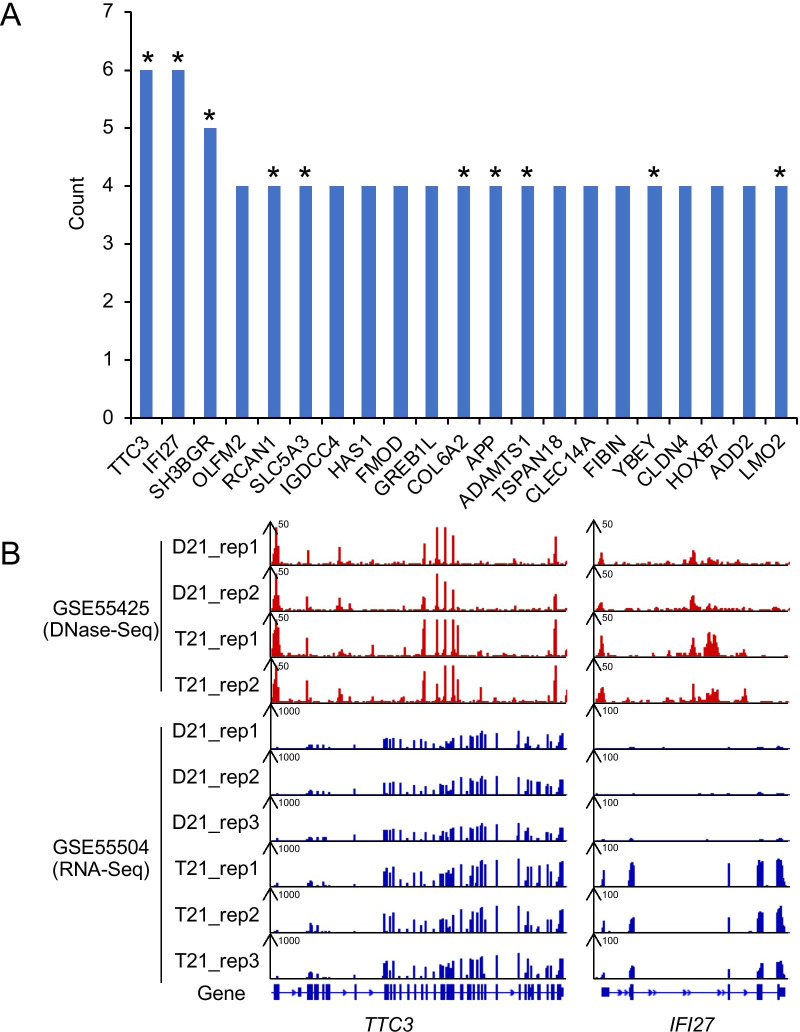
Prospective biomarkers in down syndrome. **a** A total of 21 up-regulated genes counts more than 3 in fibroblasts from 8 GSE Series records, and half of which, marked with asterisk, have been reported as prospective biomarkers for trisomy 21. **b** Gene expression patterns of *TTC3* and *IFI27* gene observed from DNase-Seq data in GSE55425 and RNA-Seq data in GSE55504. T21 and D21 represent trisomy 21 and euploid controls, respectively

To discover useful clues for diseases using the BDdb database, we targeted Down syndrome in humans, which has drawn considerable attention worldwide over many years. Taking fibroblasts as an example, a total of 13 GSE Series records were linked to trisomy 21, including various sequencing types such as RNA-Seq and DNase-Seq. We consolidated the up-regulated DEGs from eight GSE Series records obtained by RNA-Seq and DNA microarray, and then sorted them by counts. In total, 21 genes had counts ≥ 4 (Fig. 4a). Among them, the *TTC3* (*tetratricopeptide repeat domain 3*) and *IFI27* (*interferon α-inducible protein 27*) genes ranked first, with counts of six. *TTC3* is located on 21q22.2 within the Down syndrome critical region (DSCR) and plays an essential role in neural development. *TTC3* is commonly regarded as a candidate gene for Down syndrome and Alzheimer’s disease [37, 38]. In addition, *IFI27* is involved in the interferon response in trisomy 21 [39]. We found that both *TTC3* and *IFI27* had higher expression levels in trisomy 21 than in euploid controls in GSE55504. This was in accordance with the chromatin accessibility pattern in GSE55425 as assessed by DNase-Seq (Fig. 4b), implying that the extra copy of chromosome 21 or other transcript regulators in Down syndrome may confer this difference. In addition to *TTC3* and *IFI37*, other eight genes (marked with asterisks in Fig. 4a) such as *SH3BGR* [40, 41] and *APP* [42] are also typical biomarkers for Down syndrome. As these well-studied trisomy 21 marker genes can be captured by the BDdb, the rest that has not been reported yet, such as *OLFM2* and *HAS1*, may be prospective biomarkers for trisomy 21. Taken together, we can theoretically seek additional biomarkers associated with a particular disease using the BDdb.

### Future perspectives

To assist clinicians and researchers, we developed the BDdb, which consists of multi-omics data and potential biomarkers of birth defects. The database will be updated constantly according to the frequency of publications associated with chromosomal aberrations. Aside from existing data, we will also add proteomics data to expand the repository. Moreover, other species and diseases will be added to provide more information to users. Ultimately, we hope that the BDdb, serving as an auxiliary tool, can provide clues for studies on birth defects, and hopefully, accelerate research progress.

## Conclusions

The BDdb is a practical tool for those in the research community committed to studying birth defects. To the best of our knowledge, the BDdb is the first comprehensive database focusing on the collection of birth-defect multi-omics data, which were curated and re-analyzed before inclusion. Users can select data of interest to investigate related diseases, which can then be retrieved and downloaded. In addition, information on DEGs, enriched GO terms and KEGG pathways, and interaction networks are provided. Notably, users can also utilize the genome browser to compare diverse samples as well as the data from various sequencing methods, e.g., RNA-Seq, ChIP-Seq, and DNA methylation, and can further explore their correlations.

## Supplementary Information

**Additional file 1: Table S1.** The basic information of collected datasets.

**Additional file 2: Table S2.** The summary of biomarkers related to birth defects.

## Data Availability

The BDdb is freely available to all users at http://t21omics.cngb.org. This web portal is accessible by web browser. The datasets used and analysed during the current study are publically available on GEO database (https://www.ncbi.nlm.nih.gov/geo/). Detailed information can be found in Additional files 1 and 2: Tables S1 and S2.

## Abbreviations

BDdb: Birth defect multi-omics database
GEO: Gene expression omnibus
SRA: Sequence read archive
ScRNA-Seq: Single-cell RNA sequencing
BS-Seq: Bisulfite sequencing
RRBS-Seq: Reduced-representation bisulfite sequencing
ChIP-Seq: Chromatin immunoprecipitation sequencing
DNase-Seq: DNase I hypersensitive sites sequencing
smRNA: Small RNA
PCGs: Protein-coding genes
lncRNAs: Long non-coding RNAs
PCR: Polymerase chain reaction
GO: Gene ontology
KEGG: Kyoto encyclopedia of genes and genomes
DEGs: Differentially expressed genes
